# Prediction of modal parameters for thin-walled blade milling process considering material removal effect

**DOI:** 10.1371/journal.pone.0323871

**Published:** 2025-09-09

**Authors:** Yu Li, Feng Ding, Weijun Tian, Dazhen Wang, Jinhua Zhou

**Affiliations:** 1 School of Mechatronic Engineering, Xi’an Technological University, Xi’an, China; 2 School of Intelligent Manufacturing and Control Technology, Xi’an MingDe Institute of Technology, Xi’an, China; 3 Engineering Practice Training Center, Northwestern Polytechnical University, Xi’an, China; 4 Xi’an Aerospace Propulsion Testing Technology Research Institute, Xi’an, China; 5 School of Mechanical Engineering, Northwestern Polytechnical University, Xi’an, China; IIIT Kurnool: Indian Institute of Information Technology Design and Manufacturing Kurnool, INDIA

## Abstract

Accurate prediction of time-varying dynamic parameters during the milling process is a prerequisite for chatter-free cutting of thin-walled parts. In this paper, a matrix iterative prediction method based on weighted parameters is proposed for the time-varying structural modes during the milling of thin-walled blade structures. The thin-walled blade finite element model is established based on the 4-node plate element, and the time-varying dynamic parameters of the workpiece during the cutting process can be obtained by modifying the thickness of the nodes through the constructed mesh element finite element model It is not necessary to re-divide the mesh elements of the thin-walled parts at each cutting position, thus improving the calculation efficiency of the dynamic parameters of the workpiece. To further improve the prediction accuracy and efficiency of the finite element model, a three-layer neural network model is constructed, which takes the calculation results of the finite element model of the plate element as training samples to obtain the neural network model. The neural network model achieves a maximum prediction error of 2.02% compared to the finite element benchmark. Meanwhile, the training time of the three-layer neural network model is about 12 seconds. When the training model is used to batch calculate the dynamic parameters of the workpiece in different cutting stages, the loading time of the model and input data is about 1.2876s, and when the number of predicted cutting stage is 360, the prediction time is only 0.0039s. Using three-layer neural network model on the premise of ensuring the calculation accuracy can greatly improve the calculation efficiency.

## 1. Introduction

In recent years, more and more novel materials have appeared in advanced manufacturing, such as abaca fibre-reinforced composites [1] and SiC/TiO2-reinforced aluminium MMCs [2]. However, in aerospace manufacturing titanium alloy thin-walled blades still are critical yet challenging components due to their susceptibility to chatter during finishing. Existing methods struggle to adapt to time-varying dynamics caused by material removal. Recent studies on hybrid composite structures, such as carbon/basalt-reinforced sandwich plates with PET foam cores, have demonstrated the importance of balancing lightweight properties and dynamic stability through combined finite element analysis and experimental validation [3,4], highlighting the need for efficient modeling techniques in time-varying systems. In order to improve the surface finish quality, it is necessary to suppress the chatter during cutting. Optimizing the cutting parameters by constructing a milling stability lobe diagram is an effective way to suppress cutting chatter. However, it should be noted that in the finishing stage of thin-walled parts, the dynamic parameters of the workpiece will change significantly with the removal of material. Therefore, in order to improve the accuracy of the stability lobe diagram prediction, the influence of the material removal process on the stability lobe diagram must be considered. The prediction methods of time-varying modal parameters in the cutting process have been extensively studied by scholars at home and abroad.

Song et al. [5] used the Sherman-Morrison-Woodbury equation to predict the frequency response functions (FRFs) of the workpiece during the milling of thin-walled parts. Meshreki et al. [6] proposed an analytical model for calculating the dynamic parameters of thin-walled parts during milling considering the variation of the workpiece thickness. Ahmadi [7] used FSMs (Finite Strip Models) to obtain the dynamic parameters of the workpiece during frame structure milling. Bravo et al. [8] pointed out that the variation of workpiece dynamic parameters affects the milling stability limit of thin-walled parts, and they obtained the dynamic parameters of thin-walled parts under different cutting steps by using modal tests. Arnaud et al. [9] analyzed the variation of workpiece dynamic parameters during the cutting process through finite elements and studied the influence of material removal and cutting position on the cutting stability boundary. Tuysuz et al. [10] proposed a process model for updating workpiece dynamics parameters in the frequency domain based on full-order and reduced-order dynamics substructures. However, it is very time-consuming to perform frequent repetitive tests on the workpiece during the cutting process, so the researchers proposed to use numerical methods to study the influence of changes in the workpiece dynamics parameters on the stability of the milling process. Thevenot et al. [11] studied the variation of workpiece dynamic parameters during machining by combining experimental testing and finite element modeling. Seguy et al. [12] used the Finite Element Method (FEM) to study the evolution of workpiece dynamics parameters during the milling of thin-walled parts. Song et al. [13] first used the experimental data to correct the finite element calculation values, and then used the corrected finite element model to establish the cutting dynamics equations considering the time-varying nature of the workpiece dynamic parameters. The advantage of this method is that the prediction accuracy can be ensured as long as the boundary conditions of the model are correctly defined. However, the reconstruction of the finite element model and the recalculation of the modal analysis are very time-consuming. In order to avoid frequent modeling and meshing of the workpiece finite element model and improve the calculation efficiency of the dynamic parameters of the workpiece, researchers have proposed a variety of algorithms.

Song et al. [14] proposed a spatio-temporal discrete method for predicting the milling stability of thin-walled parts based on the thin-plate theory and modal superposition principle, which takes into account the influence of the tool-workpiece contact position and the multimodal state of the system. Tian, Ge, and Zhao [15–17] conducted modal tests to investigate the modal parameters when cutting thin-walled parts. Adetoro et al. [18] analyzed the dynamic properties of the workpiece using the finite element method. Alan, Budak, Wang and Yang et al. [19–22] predicted the dynamic frequency function of the workpiece by using the structural dynamics modification method based on finite element analysis. Shi and Wang et al. [23,24] conducted an in-depth study on the theory of dynamic properties, proposed a multimodal coupling method comprehensively considering modal effects, and established a new calculation model to study the time-varying problem of the dynamic characteristics of the workpiece. It is time-consuming and laborious to carry out frequent modal tests on the workpiece during the cutting process by using the modal experiment method, but the use of finite element simulation improves the simulation efficiency and greatly promotes the application of numerical simulation technology in the stability of milling. However, when the structural parameters of thin-walled parts in the cutting process change frequently and slightly during the removal process, the finite element simulation is obviously time-consuming and laborious..In the literature [20], three-dimensional cubic elements are used to conduct finite element modeling for thin-walled blades, which will lead to a large degree of freedom of the model and it is very time-consuming to simulate and analyze the cutting process by using finite element software. The method proposed in the literature [25] needs to assume the vibration equation satisfied by the workpiece according to the boundary conditions. If the form of the assumed vibration equation is unreasonable, and the calculation error is large, the maximum error of the first two orders of the modal natural frequency obtained by the solution of this study is 8%. In the literature [26], the matrix perturbation method is used to predict the natural frequency of the blade structure and the maximum error is 7.3%, which is a large error.

Deep learning technology is widely used in dynamic parameter prediction, while traditional methods such as finite element analysis remain critical for validating composite behaviors. For instance, studies on bio-composite skew plates [27] and natural fiber-reinforced sandwich structures [26] have shown that FSDT-based models combined with experimental data can effectively capture frequency responses, providing a foundation for hybrid approaches in dynamic modeling. In the field of CNC machining, it has been applied to cutting force prediction, tool wear prediction, and workpiece surface roughness prediction [28–32]. To improve the real-time prediction capability of time-varying dynamic parameters for milling thin-walled parts, this paper applies deep learning technology to dynamic parameter prediction. Further, a stable cutting parameter domain can be provided for modelling and optimizing the residual stresses in multi-axis machining of thin-walled blades [33,34].

In this paper, firstly, the finite element method is used to discretize the thin-walled structure and obtain the mass matrix and stiffness matrix of the structure. Then, the dynamic characteristics of thin-walled parts after modification are solved based on the matrix iteration method. Finally, a three-layer neural network model for the prediction of time-varying dynamic parameters of thin-walled parts milling is constructed and the natural frequency after removing the small amplitude adjustment of structural parameters is calculated accordingly..The results obtained from finite element simulation and modal testing are compared with those obtained from calculations, which show that the method of structural dynamic correction using the matrix iteration method is effective and reliable in dynamic modal prediction during CNC machining of thin-walled parts, laying a foundation for rapid establishment of three-dimensional stability and the prediction of optimal milling parameters.

## 2. Evolution and solution of dynamic characteristics of thin-walled blade milling

### 2.1 Discretization of thin-walled blade geometry

**Fig 1:**
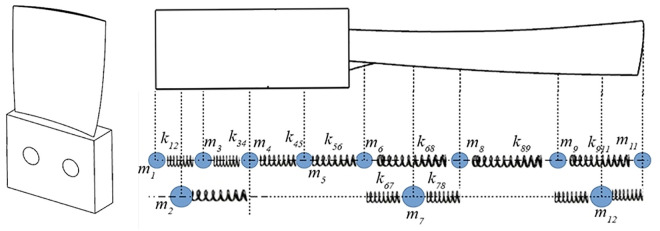
Simplification and discretization of the blade process model.

The aerospace blade structure is characterized as a typical thin-walled part with a cross section that varies greatly in thickness along the chord length direction, which makes it difficult to characterize its structural properties with specific geometric parameters. To analyze this continuous medium dynamics problem, the idea of finite element [35] should be adopted to discretize its spatial domain and obtain a discretized multi-degree-of-freedom dynamics model, as shown in Fig 1.

For the discretized model of the blade, its structural dynamics equation in the physical coordinate system can be expressed as:


$$M\ddot{u}+C\dot{u}+Ku=0$$ (1)

Where u, $\dot{u}$ and $\ddot{u}$ represent the displacement vector, velocity vector and acceleration vector respectively; and M, C and K represent the mass matrix, damping matrix and stiffness matrix of the workpiece, respectively.

### 2.2 Evolution of dynamic parameters of blade milling process

**Fig 2:**
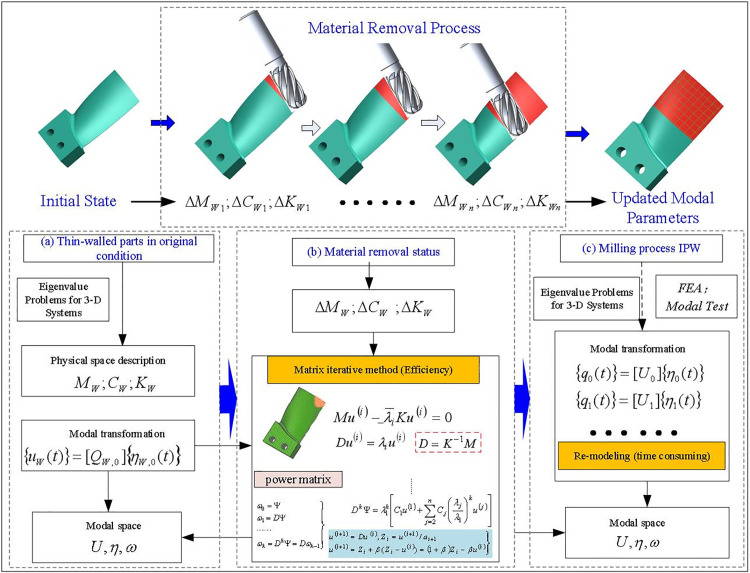
Evolution of dynamic characteristics of thin-walled blade milling.

During the milling of thin-walled parts, material removal significantly impacts the dynamic characteristics of the entire machining system. Therefore, in the prediction of the stable domain, it is necessary to be able to predict in real time the dynamic structural characteristics of the thin-walled parts after the cutting state of each position is changed, and update the characteristics of the workpiece in the stable domain model, as shown in Fig 2.

#### (1) Initial state of the first phase.

Thin-walled parts are in semi-finishing state at the beginning of milling. In the milling process system composed of cutter-workpiece, the dynamic equation of the, thin-walled parts process subsystem can be expressed in matrix form as follows:


$$\left[M_{w,s}\mathrm{\backslash rightleft}\{\ddot{u}(t)\right\}+\left[C_{w,s}\right]\left\{\dot{u}(t)\right\}+\left[K_{w,s}\right]\left\{u(t)\right\}=\left\{F_{w,s}\right\}$$ (2)

In order to show the dynamic characteristics of each part of the thin-walled parts, the thin-walled parts need to be discretized into a finite number of grid elements composed of the continuum with reference to subsection 2.1.Then u(t) is the displacement vector of the grid node, each node contains three degrees of freedom *x-y-z*, then $\left[M_{W,S}\right]$, $\left[C_{W,S}\right]$ and $\left[K_{W,S}\right]$ repectively represent the mass matrix, damping matrix and stiffness matrix of the thin-walled parts at the beginning of the machining process, i.e., at the semi-finishing state. $F_{W,S}$ is the cutting force applied to the workpiece, and the “*S*” subscript indicates the initial state.

The vibration displacement solution of the above dynamics equation corresponds to the undamped free vibration of the workpiece subsystem, whose equation can be expressed as:


$$\left[M_{w,s}\mathrm{\backslash rightleft}\{\ddot{u}(t)\right\}+\left[K_{w,s}\right]\left\{u(t)\right\}=0$$ (3)

Then the eigenvalues of the physical space of the workpiece subsystem can be expressed as:


$$(K_{W,S}-\lambda M_{W,S})Q_{W}(t)=0$$ (4)

By solving the above equations, the frequency and mode shapes of each order of the workpiece subsystem can be obtained. In the analysis of practical problems, the first 2 ~ 4 orders of frequency modes are often intercepted for analysis and calculation, so the modal matrix should be smaller than 3 × 4 matrix. The dynamic equation can be transformed from physical space to modal space by modal transformation:


$$Q_{W,S}^{T}M_{W,S}Q_{W,S}\left\{{\ddot{\eta }}_{W,S}\right\}+Q_{W,S}^{T}K_{W,S}Q_{W,S}\left\{\eta_{W,S}\right\}=0$$ (5)

Where $\left\{\eta_{W,0}(t)\right\}$ represents the coordinate vector in modal space.

Substitute Eq. (5) into Eq. (3) and the modal space dynamics equation is obtained by the left multiplificative modal matrix ${\left[Q_{W,0}\right]}^{T}$.


$$Q_{W,S}^{T}M_{W,S}Q_{W,S}\left\{{\ddot{\eta }}_{W,S}\right\}+Q_{W,S}^{T}K_{W,S}Q_{W,S}\left\{\eta_{W,S}\right\}=0$$ (6)

According to the orthogonality condition of the modal matrix:


$$\left\{ \begin{array}{c} Q_{W,S}^{T}M_{W,S}Q_{W,S}=I\\ Q_{W,S}^{T}K_{W,S}Q_{W,S}=\omega_{W,S}^{2} \end{array}\right.$$ (7)

Then after modal transfromation, the damping ratio matrix of thin-walled blade can be obtained by modal experiments when damping is considered. Its dynamic equation can be further expressed as:


$${\ddot{\eta }}_{W,s}(t)+2\xi_{w,s}\omega_{W,s}\dot{\eta }(t)+\omega_{W,s}^{2}\eta (t)=\left[Q_{W,s}^{T}\right]F_{w,s}$$ (8)

#### (2) Status of the phase II IPW.

In the process of thin-walled parts milling, due to the sensitivity of the structure, the material removal process will lead to significant changes in mass, damping and stiffness of the workpiece. In order to solve the modal parameters in the process, the unit removal quantities of the process are respectively represented as: $\Delta M_{IPW,m}$, $\Delta K_{IPW,m}$, then the dynamic equation of the undamped free vibration system can be expressed as:


$$\left(M_{w,s}+\Delta M_{IPW,m}\right)\ddot{u}(t)+\left(K_{w,s}+\Delta K_{IPW,m}\right)u(t)=0$$ (9)

By performing the spatial modal transformation through equation (5), the above equation can be expressed as:


$$Q_{W,S}^{T}\left(M_{W,S}+\Delta M_{IPW,m}\right)Q_{W,S}\left\{{\ddot{\eta }}_{W,S}\right\}+Q_{W,S}^{T}\left(K_{W,S}+\Delta K_{IPW,m}\right)Q_{W,S}\left\{\eta_{W,S}\right\}=0$$ (10)

Substitute Eq. (7) into Eq. (10) to get the equation as follows:


$$\left(I+Q_{W,S}^{T}\Delta M_{W,S}Q_{W,S}\right){\ddot{\eta }}_{W,S}+\left(\omega_{W,S}^{2}+Q_{W,S}^{T}\Delta K_{W,S}Q_{W,S}\right)\eta (t)=0$$ (11)

The modal parameters in the process can be solved by solving the characteristic equation of the above equation, i.e., the characteristic equation of Eq. (11) can be further expressed as:


$$Q_{W,S}^{T}M_{W,S}Q_{W,S}\left\{{\ddot{\eta }}_{W,S}\right\}+Q_{W,S}^{T}K_{W,S}Q_{W,S}\left\{\eta_{W,S}\right\}=0$$ (12)

The eigenvalue solution of this equation includes a new natural frequency and spatial transformation:


$$\eta W,S=Q_{IPW}\eta W,IPW$$ (13)

Where $Q_{IPW}$ is the modal matrix corresponding to the new natural frequency $\omega_{W,IPW}$. Substitute equation (13) into equation (5), the modal matrix during milling can be written in the form of the product of the initial modal matrix and the transformation matrix, as follows:


$$Q_{W,m}=Q_{W,S}Qm$$ (14)

Similarly, the dynamics equation of the workpiece at the m th tool position can be expressed in the modal space as:


$${\ddot{\eta }}_{W,m}(t)+2\xi_{W,m}\omega_{W,m}\dot{\eta }(t)+\omega_{W,m}^{2}\eta (t)=Q_{W,m}^{T}F_{W,m}(t)$$ (15)

$Q_{W,m}$ is the complete modal matrix during milling. In the stability prediction of thin-walled parts, only the modal matrix ${\hat{Q}}_{W,m}$ corresponding to the region nodes, which is part of the complete matrix, is modified each time. The detailed solution procedure is discussed in Section 2.3.

Therefore, a modeling method based on the structural dynamic correction strategy of the matrix iteration is proposed in this section to solve the time-varying dynamic characteristics of thin-walled parts during milling. The key advantage of this method is that only the initial dynamic modeling and parameter calculation of the workpiece are required to obtain both the dynamic parameters of the workpiece during the cutting process at any tool position in the milling process. In the process of execution, the subsequent results are obtained through the dynamic modification of the initial workpiece dynamic parameter structure, and there is no need to re-model the cutting process or repeat the modal experimental analysis. The proposed basic idea is shown in Fig 2, which is detailed below.

### 2.3 Dynamic parameter solution based on matrix iteration method

When the damping of the workpiece is ignored, the eigenvalue problem of Eq. (1) is formulated as a modal space, which can be expressed as:


$$Mu^{\left(i\right)}={\bar{\lambda }}_{i}Ku^{\left(i\right)}$$ (16)

Eq. (16) is a generalized eigenvalue problem that can be transformed to a standard eigenvalue problem by introducing the power matrix $D=K^{-1}M$, that is:


$$Du^{\left(i\right)}=\lambda_{i}u^{\left(i\right)}$$ (17)

In Eq. (17), of the eigenvalue $\lambda_{i}=\frac{1}{\omega_{i}^{2}}$, $\omega_{i}$ is the natural frequency of the thin-walled part, and $u^{\left(i\right)}$ is the eigenvector corresponding to the eigenvalue μ.For the vibration problem, the corresponding natural frequency and modal vector can be obtained by solving the eigenvalues and eigenvectors in Eq. (17).

Choose a hypothetical mode Ψ of the system, which is generally not a real mode but can always be expressed as a linear combination of real modes:


$$\Psi =C_{1}u^{\left(1\right)}+C_{2}u^{\left(2\right)}+\cdots C_{n}u^{\left(n\right)}=\sum {\mathrm{\backslash nolimits}}_{j=1}^{n}C_{j}u^{\left(i\right)}=uC$$ (18)

Where $u=\left[\begin{array}{cc} {}*20c\begin{array}{cc} {}*20cu^{\left(1\right)} & u^{\left(2\right)} \end{array} & \begin{array}{cc} {}*20c\cdots & u^{\left(n\right)} \end{array} \end{array}\right]$ is the modal matrix, $C={\left[\begin{array}{cc} {}*20c\begin{array}{cc} {}*20cC_{1} & C_{2} \end{array} & \begin{array}{cc} {}*20c\cdots & C_{n} \end{array} \end{array}\right]}^{T}$. Eq. (18) is obtained by left multiplication of the power matrix D:


$$D\Psi =\sum_{j=1}^{n}C_{j}Du^{\left(j\right)}=\sum_{j=1}^{n}C_{j}\lambda_{j}u^{\left(j\right)}=\lambda_{1}\left[C_{1}u^{\left(1\right)}+\sum_{j=2}^{n}C_{j}\frac{\lambda_{j}}{\lambda_{1}}u^{\left(j\right)}\right]$$ (19)

The equation below can be obtained by left multiplication of the power matrix D based on the above equation:


$$D\left(D\Psi \right)=D^{2}\Psi =\lambda_{1}^{2}\left[C_{1}u^{\left(1\right)}+\sum_{j=2}^{n}C_{j}{\left(\frac{\lambda_{j}}{\lambda_{1}}\right)}^{2}u^{\left(j\right)}\right]$$ (20)

After *K* times of left-multiplication of the D matrix (equivalent to *k* iterations):


$$D^{k}\Psi =\lambda_{1}^{k}\left[C_{1}u^{\left(1\right)}+\sum_{j=2}^{n}C_{j}{\left(\frac{\lambda_{j}}{\lambda_{1}}\right)}^{k}u^{\left(j\right)}\right]$$ (21)

Since $\frac{\lambda_{j}}{\lambda_{1}}<1$, the dominance of the first term in the parentheses of the upper equation is strengthened with each iteration. The higher the number of iterations, the smaller the proportion of modal components above first order contained in the second term in the parentheses of the above equation.

Take $D^{k}\omega$ as the *k*th approximation of the first-order mode, represented by $\omega_{k}$, the matrix iteration method is as follows:


$$\begin{array}{c} \omega_{0}=\Psi \\ \omega_{1}=D\Psi \\ \cdots \cdots \\ \omega_{k}=D^{k}\Psi =D\omega_{k-1} \end{array}\}$$ (22)

If the number of iteration *k* is large enough that the remaining higher-order modal components other than the first-order modes are less than the allowable error, it can be omitted to get:


$$\omega_{k}=D^{k}\Psi =\lambda_{1}^{k}C_{1}u^{\left(1\right)}$$ (23)

Thus, after *K* iterations, the mode is approximately equal to the first order real mode.

One more iteration for $\omega_{k}$ to get:


$$\omega_{k+1}=D\omega_{k}=\lambda_{1}\omega_{k}$$ (24)

For the jth element $\omega_{j\left(k\right)}$ and $\omega_{j\left(k+1\right)}$ in $\omega_{k}$ and $\omega_{k+1}$, the relationship is as follows:


$$\omega_{j,k+1}=\lambda_{1}\omega_{j,k}$$ (25)

From the above equation, the fundamental frequency is:


$$\omega_{1}=\frac{1}{\sqrt{\lambda_{1}}}=\sqrt{\frac{\omega_{j\left(k\right)}}{\varpi_{j\left(k+1\right)}}}$$ (26)

After the first-order mode and fundamental frequency of the system are obtained by matrix iteration method, obtain the second-order mode and frequency with the same method.

Choose any hypothetical mode Ψ,the first-order mode $u^{\left(1\right)}$ can always be calculated by iteration. If it is assumed that the coefficient of the first-order mode in the hypothetical mode $u^{\left(1\right)}$ is $C_{1}=0$_,_the result of the iteration will be:


$$D^{k}\Psi =\sum_{j=2}^{n}C_{j}\lambda_{j}^{k}u^{\left(j\right)}=\lambda_{2}^{k}\left[C_{2}u^{\left(2\right)}+\sum_{j=3}^{n}C_{j}{\left(\frac{\lambda_{j}}{\lambda_{2}}\right)}^{k}u^{\left(j\right)}\right]$$ (27)

The result of the above equation tends to the second order mode $u^{\left(2\right)}$. Similarly, to obtain the third order mode $u^{\left(3\right)}$, $C_{1}=C_{2}=0$ must be assumed for the mode Ψ.

According to the principle of matrix iteration method above, the first trial vector is denoted as $u^{\left(0\right)}$, and D is acted on it, and $Du^{\left(0\right)}=u^{\left(1\right)}$, $u^{\left(0\right)}$ is obtained after normalization, i.e.,: the element with the largest absolute value in $u^{\left(0\right)}$ is $a_{1}$, and then $u^{\left(1\right)}/a_{1}$ is assigned to $u^{\left(1\right)}$, thus the basic format of matrix iterative method can be obtained:


$$\begin{array}{c} u^{\left(i+1\right)}=Du^{\left(i\right)}\\ u^{\left(i+1\right)}=u^{\left(i+1\right)}/a_{i+1} \end{array}\}$$ (28)

That is, the dynamic matrix D iterates repeatedly on the vector u, With each iteration step, the eigenmode of the system is approached until $u^{\left(i\right)}\approx u^{\left(i+1\right)}$ within a certain accuracy range. at which time, $u^{\left(i+1\right)}$ is the intrinsic vibration mode of thin-walled parts, and $a_{i+1}$ is the square of the nature frequency of $\omega_{0}$.

To increase the step size of each iteration, a weighted parameter is added to Eq. (28). The iteration is formulated as:


$$\begin{array}{c} u^{\left(i+1\right)}=Du^{\left(i\right)};Z_{i}=u^{\left(i+1\right)}/a_{i+1}\\ u^{\left(i+1\right)}=Z_{i}+\beta (Z_{i}-u^{\left(i\right)})=\left(1+\beta \right)Z_{i}-\beta u^{\left(i\right)} \end{array}\}$$ (29)

The size of the weighted parameter β determines the step size of each iteration. If it is too small, the effect is not obvious; but if it is too large, the iteration may oscillate, which will slow down the convergence speed. After many iterations of simulation calculation, it is found that the effect of β value (0.7–0.8) is significant.

### 2.4 Solution of mass matrix and stiffness matrix of thin-walled parts

**Fig 3:**
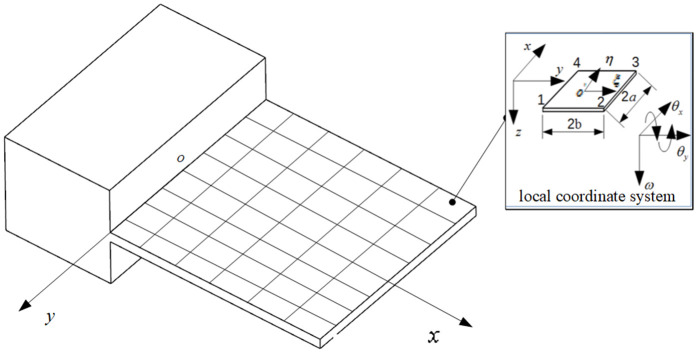
4-node plate cell meshing.

As can be seen from the process of matrix iteration method, the key to calculating the modal parameters of thin-walled parts is to solve the mass matrix and stiffness matrix. Based on the finite element method combined with the actual structural characteristics of the thin-walled blade, the blade is simplified as a thin plate. The 4-node plate element is selected to efficiently simulate the bending-dominated behavior of thin-walled structures. Compared to the 8-node solid element, the degrees of freedom can be significantly reduced without introducing errors in the first three natural frequencies. This trade-off is crucial for real-time predictions in multi-stage machining processes. Therefor the 4-node plate element is used for meshing, as shown in Fig 3.

Suppose the plate element has 1, 2, 3, and 4 nodes in the local coordinate system, and each corner node has three parameters, namely, the deflection ω, the angle of rotation $\theta_{x}$ of the normal about the x -axis, and the angle of rotation $\theta_{y}$ about the y -axis, i.e.,:


$$\{\delta_{i}\}=\{\omega_{i},\theta_{xi},\theta_{yi}{\}}^{T}={\left\{\omega_{i},{\left(\frac{\partial \omega }{\partial y}\right)}_{i},-{\left(\frac{\partial \omega }{\partial x}\right)}_{i}\right\}}^{T}$$ (30)

The plate element has 4 nodes and 12 node displacement components, then the displacement array of the whole element is ${\left\{\delta \right\}}_{12\times 1}^{e}=[\delta_{1}^{T},\delta_{2}^{T},\delta_{3}^{T},\delta_{4}^{T}{]}^{T}$. In the local coordinate system ( ηoξ), the length of the element is 2a and the width is 2b.Only take the deflection as an independent displacement component, and substitute the node coordinates and node displacements into the displacement function, then the deflection at any point in the element can be expressed by the shape function $N_{i}(x,y)$ and the element node displacement $\left\{\delta_{i}\right\}$:


$$\omega ={\left[N\right]}_{1\times 12}{{\left\{\delta \right\}}^{e}}_{12\times 1}$$ (31)

Where *N* is the interpolation function, which is expressed as follows for the plate element:


$$\begin{array}{c} N_{i}=\frac{1}{8}(1+\xi \xi_{i})(1+\eta \eta_{i})(2+\xi \xi_{i}+\eta \eta_{i}-\xi^{2}-\eta^{2})\\ N_{ix}=\frac{1}{8}b\eta_{i}(1+\xi \xi_{i})(1+\eta \eta_{i})(1-\eta^{2})\\ N_{iy}=\frac{1}{8}b\xi_{i}(1+\xi \xi_{i})(1+\eta \eta_{i})(1-\xi^{2}) \end{array}\}\;\begin{array}{c} \eta =\frac{x}{a};\eta_{i}=\frac{x_{i}}{a}\\ \xi =\frac{y}{b};\xi_{i}=\frac{y_{i}}{a} \end{array}\}$$ (32)

$K^{e}=\int_{V^{e}}B^{T}DBdV$ can be solved according to the definition of the element stiffness matrix, where D is the elasticity matrix, and


$$D=\frac{Et^{3}}{12(1-v^{2})}\left[\begin{array}{ccc} {}*20c1 & v & 0\\ v & 1 & 0\\ 0 & 0 & \frac{1-v}{2} \end{array}\right]\;{\left[B_{i}\right]}_{3\times 3}=-z\left\{\begin{array}{c} {}[N{]}_{i,xx}\\ {}[N{]}_{i,xx}\\ {}[N{]}_{i,xx} \end{array}\right\}=-z\left\{\begin{array}{c} {}[N{]}_{i,\xi \xi }/a^{2}\\ {}[N{]}_{i,xx}/b^{2}\\ {}[N{]}_{i,xx}/ab \end{array}\right\}$$ (33)

Here k will be written in block form:


$${\left[K\right]}_{12\times 12}=\left[\begin{array}{cccc} {}*20ck_{11} & k_{12} & k_{13} & k_{14}\\ k_{21} & k_{22} & k_{23} & k_{24}\\ k_{31} & k_{32} & k_{33} & k_{34}\\ k_{41} & k_{42} & k_{43} & k_{44} \end{array}\right]$$ (34)

The centralized mass matrix of the plate element is a diagonal matrix of order 12, which actually reflects four non-zero elements at the four nodes in the matrix. If the thickness of the thin plate is consistent, it is the homogeneity of the coordinated mass at the nodes. Thus, the element coordination mass matrix is expressed as:


$$M^{e}=\int_{V^{e}}\rho N^{T}NdV$$ (35)

Assuming the transformation matrix G from local to global coordinates, the stiffness matrix and mass matrix in global coordinates can be expressed as:


$$K_{o}^{e}=G^{T}K^{e}G$$

$$M_{o}^{e}=G^{T}M^{e}G$$ (36)

Thus, the global stiffness matrix and mass matrix of thin-walled parts can be expressed as:


$$K=\sum G^{T}K^{e}G=\sum G^{T}[\int_{V^{e}}B^{T}DBdV]G$$

$$M=\sum G^{T}M^{e}G=\sum G^{T}[\int_{V^{e}}\rho N^{T}NdV]G$$ (37)

After the total mass matrix and stiffness matrix of the thin-walled parts are calculated, the relevant dynamic parameters of thin-walled parts can be obtained by substituting the results into (29) for calculation.

## 3. Neural network based prediction of dynamic parameters

### 3.1 Neural network based dynamics modeling

With the development of deep learning technology, neural network has been widely used in cutting force prediction, tool life prediction and other fields. The GPU-accelerated neural network can process multiple cutting states in batches, significantly reducing computation time and demonstrating a marked advantage over finite element methods in terms of efficiency. Additionally, parallel computing helps eliminate the accumulation of serial errors, ensuring consistency in predictions.

**Fig 4:**
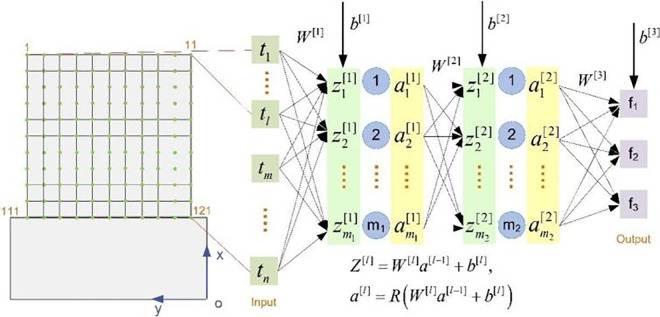
Neural network structure diagram.


In order to achieve accuracy and efficiency in solving the dynamic parameters of thin-walled parts, the three-layer neural network shown in Fig 4 is used in this paper to predict the dynamic parameters of thin-walled blade milling. Taking a single sample point as an example, the calculation process is described. The input variable t is the thickness of the element node, and the output variable f is the first three orders of the natural frequency of the workpiece, expressed as:

$$\begin{array}{c} 𝐭={\left[\begin{array}{cccc} {}*20ct_{1} & t_{2} & \cdots & t_{n} \end{array}\right]}^{T}\\ 𝐟={\left[\begin{array}{ccc} {}*20cf_{1} & f_{2} & f_{3} \end{array}\right]}^{T} \end{array}$$ (38)

The ReLU function is used for the activation function, which is expressed as:


R(x)=max(0,x) (39)

Based on the inputs t, the weight matrix $W^{\left[1\right]}$ and the bias $b^{\left[1\right]}$ of the first layer of the network, as well as the activation function, the following can be calculated:


$$\begin{array}{c} {𝐙}^{\left[1\right]}=\left[\begin{array}{cccc} {}*20cw_{1,1}^{\left[1\right]} & w_{1,2}^{\left[1\right]} & \cdots & w_{1,n}^{\left[1\right]}\\ w_{2,1}^{\left[1\right]} & w_{2,2}^{\left[1\right]} & \cdots & w_{2,n}^{\left[1\right]}\\ \vdots & \vdots & \ddots & \vdots \\ w_{m_{1},1}^{\left[1\right]} & w_{m_{1},2}^{\left[1\right]} & \cdots & w_{m_{1},n}^{\left[1\right]} \end{array}\mathrm{\backslash rightleft}[\begin{array}{c} {}*20ct_{1}\\ t_{2}\\ \vdots \\ t_{n} \end{array}\right]+{𝐛}^{\left[1\right]}={𝐖}^{\left[1\right]}𝐭+{𝐛}^{\left[1\right]},\\ {𝐚}^{\left[1\right]}=R({𝐙}^{\left[1\right]}) \end{array}$$ (40)

Based on the output $a^{\left[1\right]}$ of the first layer network, the weight matrix $W^{\left[2\right]}$ and bias $b^{\left[2\right]}$ of the second layer network as well as the activation function, the following can be calculated:


$$\begin{array}{c} {𝐙}^{\left[2\right]}=\left[\begin{array}{cccc} {}*20cw_{1,1}^{\left[2\right]} & w_{1,2}^{\left[2\right]} & \cdots & w_{1,m_{1}}^{\left[2\right]}\\ w_{2,1}^{\left[2\right]} & w_{2,2}^{\left[2\right]} & \cdots & w_{2,m_{1}}^{\left[2\right]}\\ \vdots & \vdots & \ddots & \vdots \\ w_{m_{2},1}^{\left[2\right]} & w_{m_{2},2}^{\left[2\right]} & \cdots & w_{m_{2},m_{1}}^{\left[2\right]} \end{array}\right]\left[\begin{array}{c} {}*20ca_{1}^{\left[1\right]}\\ a_{2}^{\left[1\right]}\\ \vdots \\ a_{m_{1}}^{\left[1\right]} \end{array}\right]+{𝐛}^{\left[2\right]}={𝐖}^{\left[2\right]}{𝐚}^{\left[1\right]}+{𝐛}^{\left[2\right]},\\ {𝐚}^{\left[2\right]}=R({𝐙}^{\left[2\right]}) \end{array}$$ (41)

Based on the output $a^{\left[2\right]}$ of the second layer network, the weight matrix $W^{\left[3\right]}$ and the bias $b^{\left[3\right]}$ of the third layer network, the following can be calculated:


$$𝐟=\left[\begin{array}{c} {}*20cf_{1}\\ f_{2}\\ f_{3} \end{array}\right]=\left[\begin{array}{cccc} {}*20cw_{1,1}^{\left[3\right]} & w_{1,2}^{\left[3\right]} & \cdots & w_{1,m_{2}}^{\left[3\right]}\\ w_{2,1}^{\left[1\right]} & w_{2,2}^{\left[1\right]} & \cdots & w_{2,m_{2}}^{\left[1\right]}\\ w_{3,1}^{\left[1\right]} & w_{3,2}^{\left[1\right]} & \cdots & w_{3_{1},m_{2}}^{\left[1\right]} \end{array}\right]\left[\begin{array}{c} {}*20ca_{1}^{\left[2\right]}\\ a_{2}^{\left[2\right]}\\ \vdots \\ a_{m_{2}}^{\left[2\right]} \end{array}\right]+{𝐛}^{\left[3\right]}={𝐖}^{\left[3\right]}{𝐚}^{\left[2\right]}+{𝐛}^{\left[3\right]}$$ (42)

The root mean square error loss function between the predicted value and the nominal value of the natural frequency of the workpiece is expressed as:


$$L=\frac{1}{2}{\left\|𝐟-\hat{𝐟}\right\|}^{2}$$ (43)

Thus, the prediction of milling dynamics parameters of thin-walled parts is transformed into an optimization problem:


$$\theta^{*}=\underset{\theta }{\mathrm{\backslash arg}\min}L(\theta )$$ (44)

Where the parameter θ is solved by the neural network weight matrix W and bias vector b, using the iteration of the steepest descent method.

The partial derivative of the loss function L with respect to the weight matrix W and the bias vector b is expressed as:


$$\frac{\partial L}{\partial {𝐖}^{\left[3\right]}}=(𝐟-\hat{𝐟})({𝐚}^{\left[2\right]}{)}^{T}$$ (45)

$$\frac{\partial L}{\partial {𝐖}^{\left[2\right]}}=({𝐖}^{\left[3\right]}{)}^{T}(𝐟-\hat{𝐟})({𝐚}^{\left[1\right]}{)}^{T}$$ (46)

$$\frac{\partial L}{\partial {𝐖}^{\left[1\right]}}=({𝐖}^{\left[2\right]}{)}^{T}({𝐖}^{\left[3\right]}{)}^{T}(𝐟-\hat{𝐟})(𝐭{)}^{T}$$ (47)

$$\frac{\partial L}{\partial {𝐛}^{\left[3\right]}}=𝐟-\hat{𝐟}$$ (48)

$$\frac{\partial L}{\partial {𝐛}^{\left[2\right]}}=(𝐟-\hat{𝐟}{)}^{T}{𝐖}^{\left[3\right]}$$ (49)

$$\frac{\partial L}{\partial {𝐛}^{\left[1\right]}}=(𝐟-\hat{𝐟}{)}^{T}{𝐖}^{\left[3\right]}{𝐖}^{\left[2\right]}$$ (50)

The parameters are updated iteratively using the steepest descent method:


$$\begin{array}{c} 𝐖=𝐖-\eta \frac{\partial L}{\partial 𝐖},\\ 𝐛=𝐛-\eta \frac{\partial L}{\partial 𝐛}, \end{array}$$ (51)

After the weight matrix W and the bias vector b are obtained, they are substituted into Eq. (40), Eq. (41) and Eq. (42) to calculate the natural frequency of the thin-walled parts.

### 3.2 Comparison of experimental results and error analysis

**Fig 5:**
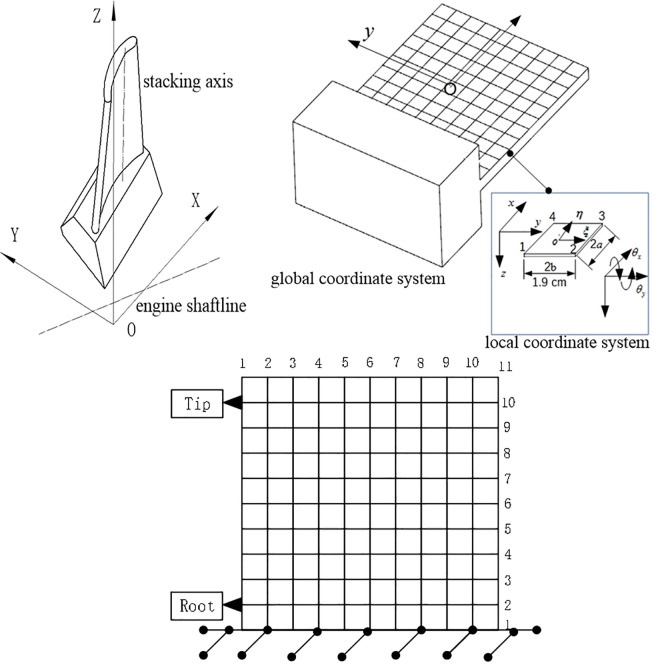
Model simplification and meshing of thin-walled parts.

The thin-walled blade is simplified into a thin plate with a length of 50 mm, a width of 50 mm and a thickness of 2 mm in accordance with the method described in Section 2.4, and it is first analyzed by the finite element method, with a 4-node plate element for meshing, and 11 nodes are set up in both the length and width directions, as shown in Fig 5. In order to simulate the clamping boundary conditions, all the degrees of freedom should be restricted at the bottom of the thin-walled workpiece, and the Lanczos method should be used for frequency extraction.

The material used in the milling process is aerospace titanium alloy TC4, with a bulk density of 4.43e-06 kg/mm^3, a Young’s modulus of 1.21e11pa, and a Poisson’s ratio of 0.34. The milling process is carried out on a JD-HGT200T machine with a maximum spindle speed of 28,000 rpm. In order to be consistent with the machining method used in the actual machining of the blades, a double-sided milling process is adopted. The thin-walled parts are clamped on the flat pliers with a 4-tooth flat-bottom milling cutter with a diameter of 10 mm. Cutting fluids are used during machining in order to maintain tool life and undamaged surface characteristics of the workpiece [36]. The spindle speed is set to 6000 rpm, the feed rate to 500 mm/min, the cutting width to 0.2 mm on one side, and cutting oil is used for cooling. In order to ensure the credibility of the test and reduce the test time, the modal test is performed on the workpieces at 6 cutting stepss: step 0 represents the initial state of the workpiece without cutting, and step 1–5 represent the materials with a cutting depth of 10 mm, 20 mm, 30 mm, 40 mm and 50 mm along the longitudinal direction, respectively.

**Fig 6:**
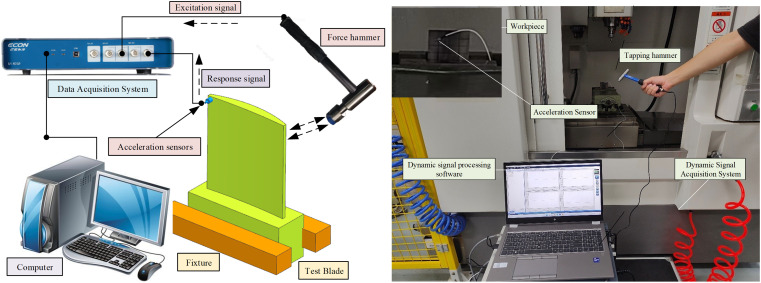
Modal testing and machining test site.

The model of force hammer is Dytran Model 5800B, the model of acceleration sensor is Dytran 3224A1, the data acquisition system is ECON-AVANT-8008, and the modal analysis software developed by Econ is used to extract the dynamic parameters of the workpiece. The testing and machining site is shown in Fig 6.

**Fig 7:**
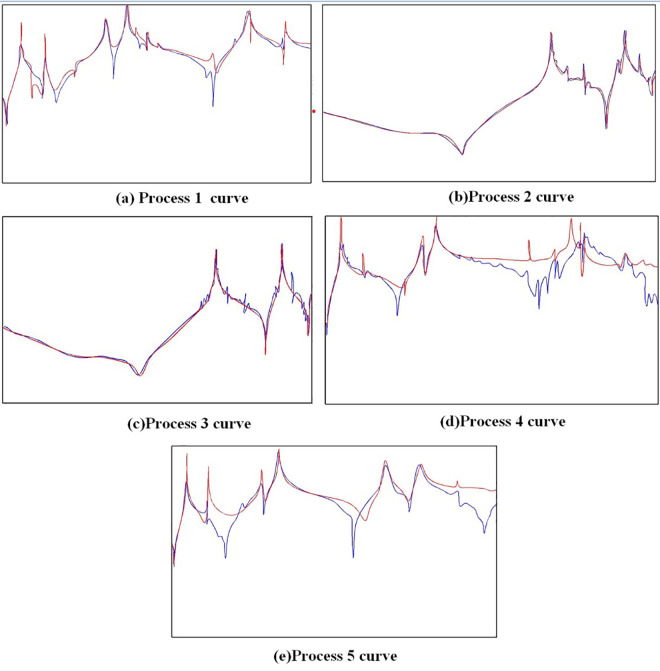
Curve of frequency response function of each process.

Through modal testing, the experimental data analyzer can extract the measured frequencies, as shown in Table 1, and obtain the frequency response function under five states, as shown in Fig 7.

**Table 1:** Natural frequency prediction and error analysis.

State	Modal order	$\omega_{A}$	$\omega_{j}$	$\omega_{s}$	$\eta_{1}$	$\eta_{2}$
Initial	first order	716.0	706.1	725.2	1.38	1.28
	second order	1708.5	1700.8	1715.6	0.45	0.42
	third order	4348.8	4309.5	4365.4	0.90	0.38
Process 1	first order	761.8	752.4	777.8	1.23	2.10
	second order	1761.1	1737.7	1784.4	1.33	1.32
	third order	4412.4	4364.6	4367.2	1.08	1.02
Process 2	first order	781.5	771.3	787.5	1.31	0.77
	second order	1727.0	1701.5	1759.5	1.48	1.88
	third order	4104.3	4055.7	4084.4	1.11	0.41
Process 3	first order	754.9	745.4	781.4	1.26	3.51
	second order	1624.1	1601.7	1673.2	1.38	3.03
	third order	3851.9	3810.0	3806.2	1.09	1.19
Process 4	first order	676.7	670.4	710.1	0.93	4.94
	second order	1513.8	1489.0	1544.3	1.64	2.01
	third order	3850.9	3797.3	3792.7	1.39	1.51
Process 5	first order	573.6	564.9	627.9	1.52	9.47
	second order	1374.0	1306.6	1504.4	4.91	9.49
	third order	3497.0	3447.6	3543.6	1.41	1.33

**Fig 8:**
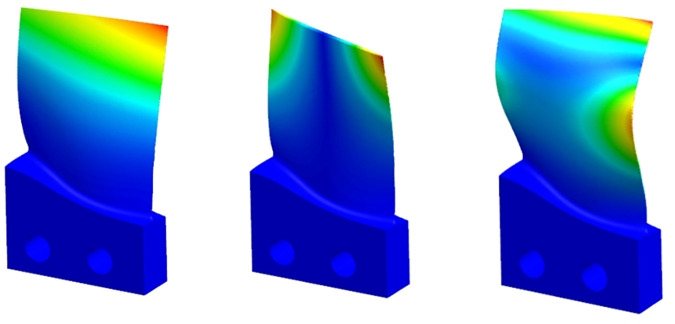
The first three orders of vibration pattern of finite element simulation.


To further illustrate the effectiveness of the matrix iteration method in this paper, a commercial finite element software is used to conduct modal analysis, which can be obtained by ABAQUS simulation. The modal pattern of order 1–3 is shown in Fig.8: The results of finite element analysis are taken as the exact value $\omega_{A}$, the results obtained by the matrix iteration method are taken as the approximate value $\omega_{j}$, and the experimental results are taken as the actual value $\omega_{S}$, then the errors can be expressed as follows.

$$\eta_{1}=\frac{\left|\omega_{A}-\omega_{j}\right|}{\omega_{A}}\times 100\%,\;\eta_{2}=\frac{\left|\omega_{A}-\omega_{s}\right|}{\omega_{A}}\times 100\%$$ (52)

The natural frequency prediction and error analysis of thin-walled parts in the material removal process are shown in Table 1.

**Fig 9:**
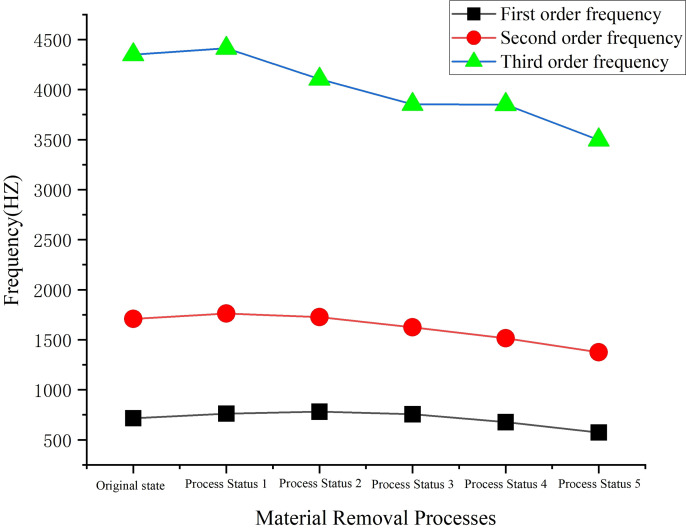
Frequency trends at different stages.

**Fig 10:**
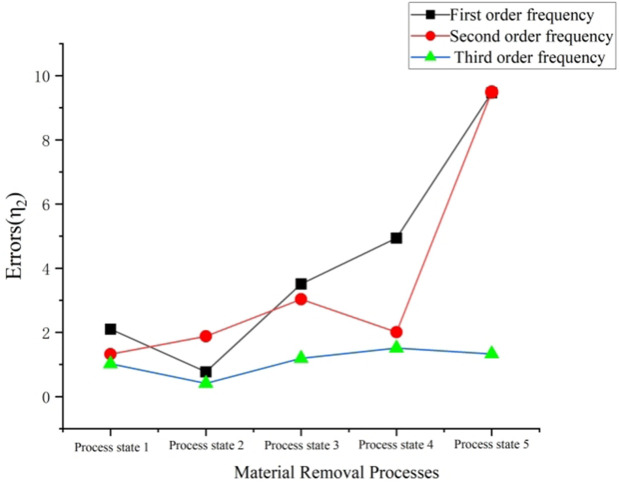
Trend graph of error change.

Through the comparison of finite element analysis, experimental modal and matrix iterative method based modal solution analysis in Table 1, it can be seen that the iterative method proposed in this paper is reliable and effective for low-order modal analysis, and it can predict the changes of dynamic parameters of each state in the material removal process in real time. Finally, according to the data in Table 1, origin is used to draw the natural frequency variation trend of each order in different process stages of the material removal process of thin-walled parts, as shown in Figs 9 and 10.

It can be seen from Fig 9 that in the milling process of thin-walled parts, the workpiece material is constantly removed, the modal stiffness and modal mass of the workpiece show an overall downward trend. In the early stage of workpiece processing, that is, from the initial workpiece to the first stage of the process, the first three orders of the frequency curve show a certain upward trend, and the decline in modal mass is dominant. With the progress of machining, the frequency of each order decreases significantly, and the modal stiffness decline is more significant.

Finally, by drawing the changing trend of $\eta_{1}$ and $\eta_{2}$, as shown in Fig 10, we can see that: the matrix iteration method has a good accuracy in calculating the changes of modal parameters of thin-walled parts in the material removal process. On the whole, the error gradually increases with the continuous removal of material. The possible reason for this phenomenon is that with the continuous removal of material, the mass of thin-walled parts decreases, and the influence of the additional mass of the sensor on the frequency gradually increases.

### 3.3 Analysis of neural network model prediction

In Section 2 of this paper, a matrix iteration method for predicting the time-varying dynamic parameters of thin-walled part milling is proposed, which only needs to obtain the geometric parameters at the nodes of discrete elements in the initial state of the workpiece to calculate the dynamic parameters of the workpiece during the cutting process. However, the iterative solution of the finite element model for each cutting state by the above method will take some time. For the discrete element structure of thin-walled parts shown in Fig 5, when the thickness t of the node is set, the first three natural frequencies of the workpiece are calculated using the 4-node plate element finite element model established in this paper, which is regarded as a sampling point. A total of 360 samples are obtained in this paper, of which 324 samples are taken as training samples.The model training learning rate is set to 0.001, the training data is scrambled, and the batch size is set to 30. Since the natural frequency of the workpiece is large relative to the thickness of the discrete unit nodes of the part, the predicted value of all samples in the same batch will be the same during the model training. Therefore, the natural frequency of the workpiece is normalized before model training and the first-order frequency is taken as an example:


$$\;f_{N}^{1}=\frac{f^{1}-f_{\min}^{1}}{f_{\max}^{1}-f_{\min}^{1}}$$ (53)

Where $f^{1}$ represents the first-order frequency, $f_{\min}^{1}$ and $f_{\max}^{1}$ respectively represent the minimum and maximum values of the first-order frequency of all samples, and $f_{N}^{1}$ represents the normalized first-order frequency.

**Fig 11:**
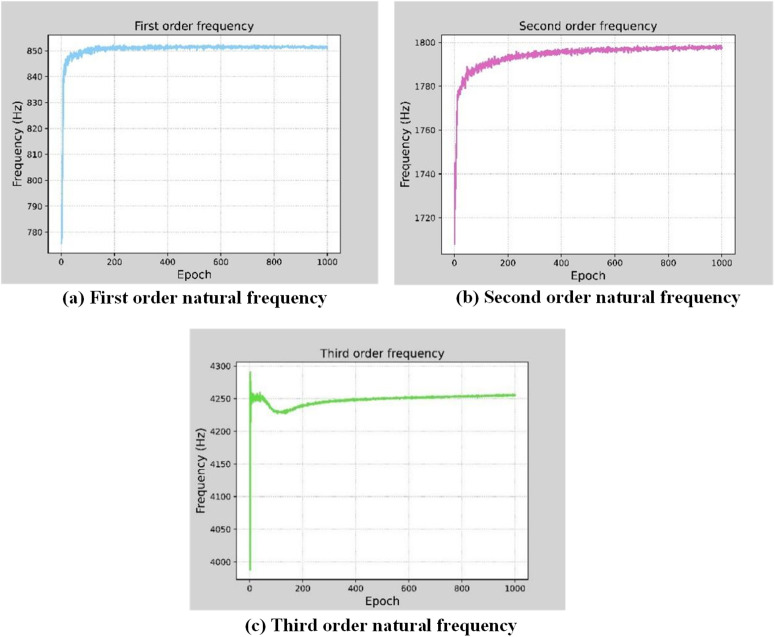
Convergence analysis of neural network model predictive values.

As the number of training rounds of the model increases, the convergence of the first three orders of the workpiece’s natural frequency is shown in Fig 11, from which we can see that the predicted value reaches convergence when epoch = 600. In this paper, epoch = 600 is used for calculation. The eigenfrequencies of the workpiece at different cutting stages calculated by the neural network model and the 4-node plate element finite element model are shown in Table 2. When the calculation results of the 4-node plate element finite element model are taken as the benchmark, the maximum error of the first three orders of the eigenfrequencies calculated by the three-layer neural network model is −2.02%. Therefore, by training the three-layer neural network model, the dynamic parameters of the workpiece during the cutting process can be effectively predicted.

**Table 2:** Comparison of the blade natural frequency (Hz) calculated by neural network (NN) model and finite element model (FEM) during cutting.

State	Modes	1	2	3	State	Modes	1	2	3
1	FEM	853.4	1809.8	4297.4	6	FEM	858.5	1798.7	4245.7
	NN	849.4	1783.1	4251.5		NN	855.8	1769.7	4197.2
	η (%)	−0.47	−1.47	−1.07		η (%)	−0.31	−1.61	−1.14
2	FEM	854.6	1807.8	4287	7	FEM	859.2	1796.3	4235.9
	NN	850.6	1780.1	4234.4		NN	855.1	1761.5	4164.4
	η (%)	−0.47	−1.53	−1.23		η (%)	−0.48	−1.93	−1.69
3	FEM	855.7	1805.7	4276.6	8	FEM	859.9	1793.7	4226.4
	NN	854.6	1779.2	4222.7		NN	856.4	1760.7	4160.5
	η (%)	−0.13	−1.47	−1.26		η (%)	−0.41	−1.84	−1.56
4	FEM	856.7	1803.4	4266.2	9	FEM	860.5	1791.1	4216.9
	NN	850.6	1776.5	4222.5		NN	856.7	1755.3	4150.9
	η (%)	−0.71	−1.49	−1.02		η (%)	−0.44	−2	−1.57
5	FEM	857.6	1801.1	4255.8	10	FEM	860.9	1788.3	4207.4
	NN	855.1	1771.9	4198		NN	856.8	1752.3	4150.9
	η (%)	−0.29	−1.62	−1.36		η (%)	−0.48	−2.02	−1.34

**Fig 12:**
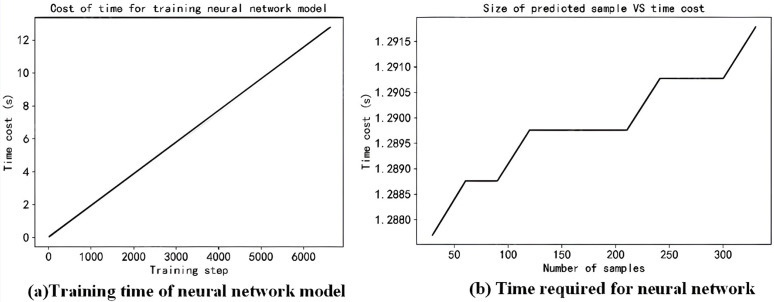
Batch prediction time of milling dynamics parameters for thin-walled parts.

Next, the calculation efficiency of the finite element model and the three-layer neural network model is compared. The computer configuration used in this paper is as follows: Processor: 12th Gen Intel(R) Core(TM) i7-12700H 2.30 GHz, Memory: 32.0 GB, Graphics Card: NVIDIA GeForce RTX 3060 Laptop GPU. The degree of freedom of stiffness matrix and mass matrix of the finite element model of thin-walled parts based on 4-node plate element is 330. The characteristic equation is solved by MATLAB software and the natural frequency of the workpiece is calculated. The time for solving one sample is 1. 504 s. When epoch = 600 and batch size = 30, the GPU is used for calculation. The training time of the three-layer neural network model is about 12s, as shown in Fig 12a. After the neural network calculation model is obtained by the training data, the model can be used to batch calculate the dynamic parameters of the workpiece at different cutting stages, and the time cost of predicting the dynamic parameters of the workpiece by the neural network model is shown in Fig 12b. The loading time of the neural network model and the model input data is about 1.2876 s, and the prediction time is only 0.0039 s when the number of samples is 360.

From the above analysis, it can be seen that compared with the 4-node plate element finite element model, the calculation efficiency can be greatly improved by using the batch calculation function of the neural network under the premise of ensuring the prediction accuracy.

## 4. Conclusion

In this paper, based on the finite element structural dynamics theory, a matrix iteration method considering weighted parameters is established for the time-varying structural modal analysis of thin-walled parts machining. The total mass matrix and stiffness matrix of the material removal process of thin-walled parts are obtained through MATLAB modeling, and the changes of modal parameters of the thin-walled parts are analyzed and predicted. Moreover, the error analysis of the prediction results is carried out by ABAQUS finite element analysis and force hammer experiment. The results show that:

(1) The proposed weighted matrix iteration method demonstrates high prediction accuracy for time-varying modal parameters during the material removal process of thin-walled parts. The evolution law of modal parameters obtained through this method facilitates the preliminary planning of machining strategies for thin-walled parts.
(2) The time-varying dynamic parameters of thin-walled parts milling are predicted by constructing a three-layer neural network. The calculation results of the 4-node plate element finite element model are used as training samples to obtain the neural network model. When the finite element model calculation results are used as the benchmark, the maximum prediction error of the neural network model is −2.02%, which ensures the prediction accuracy.
(3) At the same time, when the training sample is 324, the epoch is 600, the batch size is 30, and GPU is used for calculation, the training time of the three-layer neural network model is about 12 s. When the model is used to batch calculate the dynamic parameters of the workpiece in different cutting stages, the loading time of the neural network model and model input data is about 1.2876 s. When the number of predicted cutting states is 360, the prediction time is only 0.0039 s, which ensures the prediction efficiency.

## Supporting information

S1 File MATLAB code and modal test. (ZIP)
